# Monoclinic modification of di-*n*-butyl­dichlorido(1,10-phenanthroline-κ^2^ *N*,*N*′)tin(IV)

**DOI:** 10.1107/S1600536810048300

**Published:** 2010-11-27

**Authors:** Seik Weng Ng

**Affiliations:** a Department of Chemistry, University of Malaya, 50603 Kuala Lumpur, Malaysia

## Abstract

The Sn(IV) atom in the title compound, [Sn(C_4_H_9_)_2_Cl_2_(C_12_H_8_N_2_)], is chelated by the *N*-heterocycle; the *n*-butyl groups are *trans* to each other whereas the Cl atoms are *cis* to each other. The crystal studied was a non-merohedral twin with the minor domain being in a 15.8 (1)% proportion.

## Related literature

For the ortho­rhom­bic modification, see: Ganis *et al.* (1983 ▶).
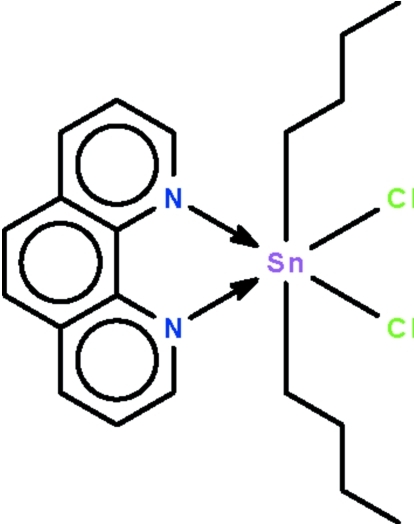


## Experimental

### 

#### Crystal data

[Sn(C_4_H_9_)_2_Cl_2_(C_12_H_8_N_2_)]
*M* *_r_* = 484.02
Monoclinic, 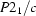

*a* = 11.1400 (2) Å
*b* = 10.4566 (2) Å
*c* = 17.9375 (4) Å
β = 92.125 (2)°
*V* = 2088.04 (7) Å^3^
*Z* = 4
Cu *K*α radiation
μ = 12.12 mm^−1^
*T* = 100 K
0.20 × 0.10 × 0.02 mm

#### Data collection

Agilent SuperNova diffractometer
Absorption correction: multi-scan (*CrysAlis PRO*; Agilent Technologies, 2010 ▶) *T*_min_ = 0.196, *T*_max_ = 0.794
15170 measured reflections
11290 independent reflections
10561 reflections with *I* > 2σ(*I*)
*R*_int_ = 0.046

#### Refinement

*R*[*F* ^2^ > 2σ(*F* ^2^)] = 0.062
*wR*(*F* ^2^) = 0.185
*S* = 1.08
11290 reflections
227 parameters
6 restraints
H-atom parameters constrained
Δρ_max_ = 2.11 e Å^−3^
Δρ_min_ = −1.95 e Å^−3^

### 

Data collection: *CrysAlis PRO* (Agilent Technologies, 2010 ▶); cell refinement: *CrysAlis PRO*; data reduction: *CrysAlis PRO*; program(s) used to solve structure: *SHELXS97* (Sheldrick, 2008 ▶); program(s) used to refine structure: *SHELXL97* (Sheldrick, 2008 ▶); molecular graphics: *X-SEED* (Barbour, 2001 ▶); software used to prepare material for publication: *publCIF* (Westrip, 2010 ▶).

## Supplementary Material

Crystal structure: contains datablocks global, I. DOI: 10.1107/S1600536810048300/hg2755sup1.cif

Structure factors: contains datablocks I. DOI: 10.1107/S1600536810048300/hg2755Isup2.hkl

Additional supplementary materials:  crystallographic information; 3D view; checkCIF report

## Figures and Tables

**Fig. 1.**
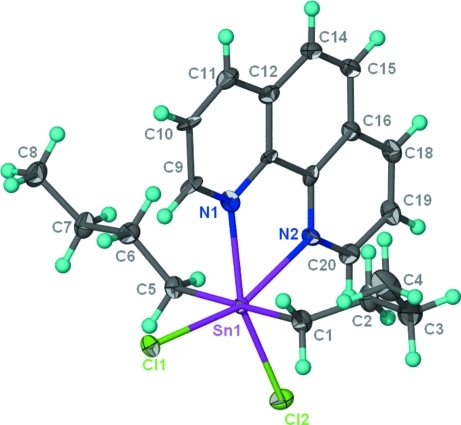
Thermal ellipsoid plot (Barbour, 2001) of SnCl2(C4H9)2(C12H8N2)2 at the 70% probability level; hydrogen atoms are drawn as spheres of arbitrary radius.

## supplementary crystallographic information

### Comment

The di-*n*-butyltin dichloride adduct with 1,10-phenanthroline (Scheme I) belongs to the *P*2_1_2_1_2_1_ space group (Ganis *et al.*, 1983). The complexation of the organotin halide with the *N*-heterocycle in ethanol solvent yielded a monoclinic modification as plate-like crystals that grew over one another. The molecule has the tin atom in an octahedral geometry (Fig. 1). The *n*-butyl groups are *trans* to each other whereas the chlorine atoms are *cis* to each other. The crystal studied is a non-merohedral twin with the minor domain being in a 15.8 (1)% proportion; the nature of the twin led to a moderately satisfactory weighting scheme in the refinement.

### Experimental

Di-*n*-butyltin dichloride (0.15 g, 0.5 mmol) and 1,10-phenanthroline hydrate (0.10, 0.5 mmol) were dissolved in boiling ethanol (10 mol). Colorless plates separated from solution after several days. A tiny platelet was prized from a lump of larger blocked that grew on top of each other.

### Refinement

Carbon-bound H-atoms were placed in calculated positions (C—H 0.95 to 0.99 Å) and were included in the refinement in the riding model approximation, with *U*(H) set to 1.2 to 1.5*U*(C). The anisotropic temperature factors of the C9 atom were restrained to be nearly isotropic so as to prevent the atom from being too oblate.

The final difference Fourier map had a peak and a hole in the vicinity of Cl2.

The crystal studied is a non-merohedral twin; the minor component refined to a 15.8 (1)% proportion.

### Crystal data

**Table:**

[Sn(C_4_H_9_)_2_Cl_2_(C_12_H_8_N_2_)]	*F*(000) = 976
*M**_r_* = 484.02	*D*_x_ = 1.540 Mg m^−^^3^
Monoclinic, *P*2_1_/*c*	Cu *K*α radiation, λ = 1.54184 Å
Hall symbol: -P 2ybc	Cell parameters from 13483 reflections
*a* = 11.1400 (2) Å	θ = 4.0–74.1°
*b* = 10.4566 (2) Å	µ = 12.12 mm^−^^1^
*c* = 17.9375 (4) Å	*T* = 100 K
β = 92.125 (2)°	Plate, colorless
*V* = 2088.04 (7) Å^3^	0.20 × 0.10 × 0.02 mm
*Z* = 4	

### Data collection

**Table:**

Agilent SuperNova diffractometer	11290 independent reflections
Radiation source: fine-focus sealed tube	10561 reflections with *I* > 2σ(*I*)
graphite	*R*_int_ = 0.046
Detector resolution: 10.4041 pixels mm^-1^	θ_max_ = 74.2°, θ_min_ = 4.0°
ω scans	*h* = −13→11
Absorption correction: multi-scan (*CrysAlis PRO*; Agilent Technologies, 2010)	*k* = −13→13
*T*_min_ = 0.196, *T*_max_ = 0.794	*l* = −22→22
15170 measured reflections	

### Refinement

**Table:**

Refinement on *F*^2^	Primary atom site location: structure-invariant direct methods
Least-squares matrix: full	Secondary atom site location: difference Fourier map
*R*[*F*^2^ > 2σ(*F*^2^)] = 0.062	Hydrogen site location: inferred from neighbouring sites
*wR*(*F*^2^) = 0.185	H-atom parameters constrained
*S* = 1.08	*w* = 1/[σ^2^(*F*_o_^2^) + (0.1271*P*)^2^ + 6.6598*P*] where *P* = (*F*_o_^2^ + 2*F*_c_^2^)/3
11290 reflections	(Δ/σ)_max_ = 0.001
227 parameters	Δρ_max_ = 2.11 e Å^−^^3^
6 restraints	Δρ_min_ = −1.95 e Å^−^^3^

### Fractional atomic coordinates and isotropic or equivalent isotropic displacement parameters (Å^2^)

**Table:**

	*x*	*y*	*z*	*U*_iso_*/*U*_eq_	
Sn1	0.850086 (19)	0.57324 (2)	0.236808 (12)	0.00995 (12)	
Cl1	0.77760 (8)	0.43243 (8)	0.12497 (5)	0.0141 (2)	
Cl2	1.07589 (8)	0.55970 (9)	0.24257 (5)	0.0181 (2)	
N1	0.6455 (3)	0.6191 (3)	0.26722 (16)	0.0131 (7)	
N2	0.8463 (3)	0.7174 (3)	0.33789 (17)	0.0132 (6)	
C1	0.8297 (4)	0.4069 (4)	0.3057 (2)	0.0167 (8)	
H1A	0.7486	0.3711	0.2953	0.020*	
H1B	0.8886	0.3419	0.2906	0.020*	
C2	0.8459 (4)	0.4277 (4)	0.3895 (2)	0.0204 (9)	
H2A	0.7851	0.4899	0.4056	0.024*	
H2B	0.9262	0.4650	0.4005	0.024*	
C3	0.8336 (5)	0.3035 (5)	0.4346 (3)	0.0290 (10)	
H3A	0.8984	0.2441	0.4210	0.035*	
H3B	0.8455	0.3238	0.4883	0.035*	
C4	0.7140 (5)	0.2362 (5)	0.4229 (3)	0.0308 (11)	
H4A	0.7134	0.1582	0.4531	0.046*	
H4B	0.7020	0.2138	0.3701	0.046*	
H4C	0.6492	0.2931	0.4378	0.046*	
C5	0.8563 (4)	0.7339 (4)	0.1626 (2)	0.0147 (7)	
H5A	0.9006	0.8037	0.1889	0.018*	
H5B	0.9039	0.7088	0.1194	0.018*	
C6	0.7366 (4)	0.7876 (4)	0.1328 (2)	0.0170 (8)	
H6A	0.6883	0.8148	0.1752	0.020*	
H6B	0.6916	0.7196	0.1054	0.020*	
C7	0.7538 (4)	0.9014 (4)	0.0808 (2)	0.0193 (8)	
H7A	0.8073	0.8760	0.0405	0.023*	
H7B	0.7933	0.9719	0.1092	0.023*	
C8	0.6344 (4)	0.9490 (4)	0.0463 (2)	0.0232 (9)	
H8A	0.6493	1.0215	0.0132	0.035*	
H8B	0.5819	0.9762	0.0859	0.035*	
H8C	0.5956	0.8799	0.0175	0.035*	
C9	0.5485 (3)	0.5678 (4)	0.2335 (2)	0.0143 (8)	
H9	0.5588	0.5109	0.1929	0.017*	
C10	0.4306 (3)	0.5949 (4)	0.2560 (2)	0.0165 (8)	
H10	0.3630	0.5561	0.2313	0.020*	
C11	0.4158 (4)	0.6779 (4)	0.3140 (2)	0.0185 (8)	
H11	0.3373	0.6981	0.3293	0.022*	
C12	0.5168 (4)	0.7337 (4)	0.3512 (2)	0.0146 (8)	
C13	0.6310 (3)	0.7008 (4)	0.3260 (2)	0.0129 (7)	
C14	0.5086 (4)	0.8193 (4)	0.4132 (2)	0.0178 (8)	
H14	0.4318	0.8409	0.4309	0.021*	
C15	0.6084 (4)	0.8701 (4)	0.4470 (2)	0.0176 (8)	
H15	0.6006	0.9274	0.4877	0.021*	
C16	0.7249 (4)	0.8387 (4)	0.4224 (2)	0.0144 (8)	
C17	0.7368 (4)	0.7535 (4)	0.3623 (2)	0.0127 (7)	
C18	0.8308 (4)	0.8872 (4)	0.4573 (2)	0.0163 (8)	
H18	0.8265	0.9445	0.4982	0.020*	
C19	0.9403 (4)	0.8510 (4)	0.4316 (2)	0.0178 (8)	
H19	1.0126	0.8832	0.4543	0.021*	
C20	0.9440 (4)	0.7658 (4)	0.3714 (2)	0.0154 (8)	
H20	1.0201	0.7416	0.3538	0.018*	

### Atomic displacement parameters (Å^2^)

**Table:**

	*U*^11^	*U*^22^	*U*^33^	*U*^12^	*U*^13^	*U*^23^
Sn1	0.01113 (16)	0.00941 (16)	0.00932 (16)	0.00028 (9)	0.00064 (10)	0.00029 (7)
Cl1	0.0165 (4)	0.0137 (5)	0.0122 (4)	−0.0033 (3)	0.0011 (3)	−0.0012 (3)
Cl2	0.0148 (4)	0.0177 (5)	0.0218 (5)	0.0010 (4)	0.0015 (4)	0.0016 (3)
N1	0.0161 (18)	0.0099 (16)	0.0135 (15)	−0.0038 (12)	0.0006 (13)	−0.0013 (11)
N2	0.0169 (17)	0.0113 (15)	0.0113 (14)	0.0026 (13)	0.0013 (12)	−0.0023 (11)
C1	0.023 (2)	0.0127 (17)	0.0148 (18)	0.0005 (16)	0.0005 (15)	0.0012 (14)
C2	0.026 (2)	0.021 (2)	0.0139 (19)	−0.0023 (17)	0.0005 (16)	0.0048 (14)
C3	0.035 (3)	0.031 (3)	0.021 (2)	0.011 (2)	0.0071 (19)	0.0113 (18)
C4	0.050 (3)	0.017 (2)	0.025 (2)	−0.001 (2)	0.010 (2)	0.0013 (17)
C5	0.0162 (18)	0.0140 (18)	0.0138 (16)	−0.0022 (15)	0.0006 (14)	0.0018 (13)
C6	0.0164 (19)	0.0157 (19)	0.0189 (18)	0.0007 (16)	−0.0001 (15)	0.0024 (15)
C7	0.024 (2)	0.0156 (18)	0.0183 (18)	−0.0009 (17)	−0.0046 (16)	0.0022 (16)
C8	0.029 (2)	0.019 (2)	0.022 (2)	0.0066 (18)	−0.0054 (17)	0.0017 (16)
C9	0.0026 (16)	0.018 (2)	0.0218 (19)	−0.0003 (14)	−0.0025 (14)	0.0012 (14)
C10	0.0059 (18)	0.0191 (19)	0.024 (2)	0.0057 (15)	−0.0003 (14)	0.0005 (15)
C11	0.0122 (18)	0.022 (2)	0.0215 (18)	0.0017 (16)	−0.0012 (15)	0.0043 (16)
C12	0.0131 (19)	0.0135 (18)	0.0171 (17)	0.0030 (15)	0.0006 (14)	0.0031 (14)
C13	0.0123 (19)	0.0141 (18)	0.0121 (16)	0.0076 (15)	0.0005 (14)	0.0030 (13)
C14	0.0169 (19)	0.020 (2)	0.0160 (17)	0.0069 (16)	0.0030 (14)	0.0007 (15)
C15	0.021 (2)	0.018 (2)	0.0141 (17)	0.0063 (16)	0.0021 (15)	−0.0023 (14)
C16	0.019 (2)	0.0119 (19)	0.0125 (16)	0.0018 (15)	0.0023 (14)	0.0024 (14)
C17	0.0144 (19)	0.0112 (18)	0.0122 (16)	0.0006 (14)	−0.0025 (13)	0.0042 (14)
C18	0.023 (2)	0.0127 (19)	0.0136 (16)	−0.0016 (16)	0.0001 (15)	−0.0011 (14)
C19	0.019 (2)	0.020 (2)	0.0144 (17)	−0.0057 (17)	−0.0001 (15)	−0.0028 (15)
C20	0.0112 (18)	0.017 (2)	0.0173 (17)	−0.0018 (15)	−0.0008 (14)	−0.0009 (15)

### Geometric parameters (Å, °)

**Table:**

Sn1—C5	2.146 (4)	C6—H6B	0.9900
Sn1—C1	2.150 (4)	C7—C8	1.530 (6)
Sn1—N2	2.360 (3)	C7—H7A	0.9900
Sn1—N1	2.412 (3)	C7—H7B	0.9900
Sn1—Cl2	2.5177 (9)	C8—H8A	0.9800
Sn1—Cl1	2.5931 (9)	C8—H8B	0.9800
N1—C9	1.331 (5)	C8—H8C	0.9800
N1—C13	1.371 (5)	C9—C10	1.417 (5)
N2—C20	1.323 (5)	C9—H9	0.9500
N2—C17	1.365 (5)	C10—C11	1.371 (6)
C1—C2	1.523 (6)	C10—H10	0.9500
C1—H1A	0.9900	C11—C12	1.413 (6)
C1—H1B	0.9900	C11—H11	0.9500
C2—C3	1.538 (6)	C12—C13	1.408 (5)
C2—H2A	0.9900	C12—C14	1.434 (6)
C2—H2B	0.9900	C13—C17	1.435 (5)
C3—C4	1.515 (7)	C14—C15	1.355 (6)
C3—H3A	0.9900	C14—H14	0.9500
C3—H3B	0.9900	C15—C16	1.424 (5)
C4—H4A	0.9800	C15—H15	0.9500
C4—H4B	0.9800	C16—C17	1.409 (5)
C4—H4C	0.9800	C16—C18	1.409 (6)
C5—C6	1.525 (5)	C18—C19	1.373 (6)
C5—H5A	0.9900	C18—H18	0.9500
C5—H5B	0.9900	C19—C20	1.402 (5)
C6—C7	1.528 (6)	C19—H19	0.9500
C6—H6A	0.9900	C20—H20	0.9500
			
C5—Sn1—C1	174.93 (15)	C7—C6—H6A	109.2
C5—Sn1—N2	88.76 (13)	C5—C6—H6B	109.2
C1—Sn1—N2	94.01 (13)	C7—C6—H6B	109.2
C5—Sn1—N1	92.13 (13)	H6A—C6—H6B	107.9
C1—Sn1—N1	84.85 (14)	C6—C7—C8	112.0 (4)
N2—Sn1—N1	69.83 (11)	C6—C7—H7A	109.2
C5—Sn1—Cl2	90.81 (11)	C8—C7—H7A	109.2
C1—Sn1—Cl2	93.29 (12)	C6—C7—H7B	109.2
N2—Sn1—Cl2	92.90 (9)	C8—C7—H7B	109.2
N1—Sn1—Cl2	162.40 (8)	H7A—C7—H7B	107.9
C5—Sn1—Cl1	88.91 (10)	C7—C8—H8A	109.5
C1—Sn1—Cl1	87.07 (11)	C7—C8—H8B	109.5
N2—Sn1—Cl1	160.62 (9)	H8A—C8—H8B	109.5
N1—Sn1—Cl1	91.04 (8)	C7—C8—H8C	109.5
Cl2—Sn1—Cl1	106.37 (3)	H8A—C8—H8C	109.5
C9—N1—C13	118.9 (3)	H8B—C8—H8C	109.5
C9—N1—Sn1	125.1 (3)	N1—C9—C10	122.4 (4)
C13—N1—Sn1	115.9 (2)	N1—C9—H9	118.8
C20—N2—C17	118.6 (3)	C10—C9—H9	118.8
C20—N2—Sn1	123.7 (3)	C11—C10—C9	118.8 (4)
C17—N2—Sn1	117.7 (3)	C11—C10—H10	120.6
C2—C1—Sn1	116.2 (3)	C9—C10—H10	120.6
C2—C1—H1A	108.2	C10—C11—C12	120.3 (4)
Sn1—C1—H1A	108.2	C10—C11—H11	119.9
C2—C1—H1B	108.2	C12—C11—H11	119.9
Sn1—C1—H1B	108.2	C13—C12—C11	117.5 (4)
H1A—C1—H1B	107.4	C13—C12—C14	119.1 (4)
C1—C2—C3	112.9 (4)	C11—C12—C14	123.4 (4)
C1—C2—H2A	109.0	N1—C13—C12	122.2 (4)
C3—C2—H2A	109.0	N1—C13—C17	118.0 (3)
C1—C2—H2B	109.0	C12—C13—C17	119.8 (4)
C3—C2—H2B	109.0	C15—C14—C12	121.1 (4)
H2A—C2—H2B	107.8	C15—C14—H14	119.4
C4—C3—C2	114.4 (4)	C12—C14—H14	119.4
C4—C3—H3A	108.6	C14—C15—C16	120.9 (4)
C2—C3—H3A	108.6	C14—C15—H15	119.6
C4—C3—H3B	108.6	C16—C15—H15	119.6
C2—C3—H3B	108.6	C17—C16—C18	117.8 (4)
H3A—C3—H3B	107.6	C17—C16—C15	119.7 (4)
C3—C4—H4A	109.5	C18—C16—C15	122.5 (4)
C3—C4—H4B	109.5	N2—C17—C16	122.1 (4)
H4A—C4—H4B	109.5	N2—C17—C13	118.5 (4)
C3—C4—H4C	109.5	C16—C17—C13	119.4 (4)
H4A—C4—H4C	109.5	C19—C18—C16	119.4 (4)
H4B—C4—H4C	109.5	C19—C18—H18	120.3
C6—C5—Sn1	117.2 (3)	C16—C18—H18	120.3
C6—C5—H5A	108.0	C18—C19—C20	119.0 (4)
Sn1—C5—H5A	108.0	C18—C19—H19	120.5
C6—C5—H5B	108.0	C20—C19—H19	120.5
Sn1—C5—H5B	108.0	N2—C20—C19	123.1 (4)
H5A—C5—H5B	107.2	N2—C20—H20	118.5
C5—C6—C7	111.9 (3)	C19—C20—H20	118.5
C5—C6—H6A	109.2		
			
C5—Sn1—N1—C9	94.0 (3)	C9—C10—C11—C12	−0.9 (6)
C1—Sn1—N1—C9	−81.9 (3)	C10—C11—C12—C13	0.2 (6)
N2—Sn1—N1—C9	−178.1 (3)	C10—C11—C12—C14	−178.7 (4)
Cl2—Sn1—N1—C9	−166.5 (2)	C9—N1—C13—C12	−1.0 (5)
Cl1—Sn1—N1—C9	5.1 (3)	Sn1—N1—C13—C12	−178.4 (3)
C5—Sn1—N1—C13	−88.8 (3)	C9—N1—C13—C17	179.0 (3)
C1—Sn1—N1—C13	95.3 (3)	Sn1—N1—C13—C17	1.7 (4)
N2—Sn1—N1—C13	−0.9 (3)	C11—C12—C13—N1	0.8 (5)
Cl2—Sn1—N1—C13	10.6 (5)	C14—C12—C13—N1	179.8 (4)
Cl1—Sn1—N1—C13	−177.7 (3)	C11—C12—C13—C17	−179.3 (3)
C5—Sn1—N2—C20	−86.5 (3)	C14—C12—C13—C17	−0.3 (5)
C1—Sn1—N2—C20	97.7 (3)	C13—C12—C14—C15	0.9 (6)
N1—Sn1—N2—C20	−179.2 (3)	C11—C12—C14—C15	179.9 (4)
Cl2—Sn1—N2—C20	4.2 (3)	C12—C14—C15—C16	−0.7 (6)
Cl1—Sn1—N2—C20	−169.7 (2)	C14—C15—C16—C17	−0.1 (6)
C5—Sn1—N2—C17	92.7 (3)	C14—C15—C16—C18	−178.5 (4)
C1—Sn1—N2—C17	−83.0 (3)	C20—N2—C17—C16	−1.0 (5)
N1—Sn1—N2—C17	0.0 (2)	Sn1—N2—C17—C16	179.7 (3)
Cl2—Sn1—N2—C17	−176.5 (2)	C20—N2—C17—C13	−179.9 (3)
Cl1—Sn1—N2—C17	9.6 (4)	Sn1—N2—C17—C13	0.8 (4)
N2—Sn1—C1—C2	−12.1 (3)	C18—C16—C17—N2	0.4 (5)
N1—Sn1—C1—C2	−81.4 (3)	C15—C16—C17—N2	−178.1 (3)
Cl2—Sn1—C1—C2	81.1 (3)	C18—C16—C17—C13	179.2 (3)
Cl1—Sn1—C1—C2	−172.7 (3)	C15—C16—C17—C13	0.7 (5)
Sn1—C1—C2—C3	−178.3 (3)	N1—C13—C17—N2	−1.7 (5)
C1—C2—C3—C4	−58.7 (5)	C12—C13—C17—N2	178.4 (3)
N2—Sn1—C5—C6	−90.3 (3)	N1—C13—C17—C16	179.4 (3)
N1—Sn1—C5—C6	−20.5 (3)	C12—C13—C17—C16	−0.5 (5)
Cl2—Sn1—C5—C6	176.9 (3)	C17—C16—C18—C19	0.3 (5)
Cl1—Sn1—C5—C6	70.5 (3)	C15—C16—C18—C19	178.7 (4)
Sn1—C5—C6—C7	180.0 (3)	C16—C18—C19—C20	−0.3 (6)
C5—C6—C7—C8	175.7 (3)	C17—N2—C20—C19	1.0 (6)
C13—N1—C9—C10	0.3 (6)	Sn1—N2—C20—C19	−179.7 (3)
Sn1—N1—C9—C10	177.4 (3)	C18—C19—C20—N2	−0.4 (6)
N1—C9—C10—C11	0.7 (6)		
